# Combining tensile testing and structural analysis at the single collagen fibril level

**DOI:** 10.1038/sdata.2018.229

**Published:** 2018-10-23

**Authors:** Andrew S. Quigley, Stéphane Bancelin, Dylan Deska-Gauthier, François Légaré, Samuel P. Veres, Laurent Kreplak

**Affiliations:** 1 Department of Physics and Atmospheric Science, Dalhousie University, Halifax, Canada; 2 Institut National de la Recherche Scientifique, Centre Énergie, Matériaux, Télécommunication, Varennes, Canada; 3 Department of Medical Neuroscience, Dalhousie University, Halifax, Canada; 4 School of Biomedical Engineering, Dalhousie University, Halifax, Canada; 5 Division of Engineering, Saint Mary’s University, Halifax, Canada

**Keywords:** Nanoscale biophysics, Biomaterials - proteins

## Abstract

Tensile testing to failure followed by imaging is a simple way of studying the structure-function relationship of connective tissues such as skin, tendon, and ligament. However, interpretation of these datasets is complex due to the hierarchical structures of the tissues spanning six or more orders of magnitude in length scale. Here we present a dataset obtained through the same scheme at the single collagen fibril level, the fundamental tensile element of load-bearing tissues. Tensile testing was performed on fibrils extracted from two types of bovine tendons, adsorbed on a glass surface and glued at both ends. An atomic force microscope (AFM) was used to pull fibrils to failure in bowstring geometry. The broken fibrils were then imaged by AFM for morphological characterization, by second harmonic generation microscopy to assess changes to molecular packing, and by fluorescence microscopy after incubation with a peptide probe that binds specifically to denatured collagen molecules. This dataset linking stress-strain curves to post-failure molecular changes is useful for researchers modelling or designing functional protein materials.

## Background & Summary

Collagen fibrils are the load-bearing element of connective tissues and a source of inspiration for researchers trying to emulate their mechanical function for tissue or material engineering purposes. The main structural features of collagen fibrils, tropocollagen molecules with a quarter-stagger arrangement^1,2^ and 65–67 nm D-band periodicity, are well conserved across a wide range of animals and tissue type^3^. Our structural understanding of the collagen fibril’s mechanical properties is typically analyzed in term of these conserved features with little attention to potential fibril to fibril variations, tissue source, or animal specificities^4^.

In this context, datasets combining mechanical testing with structural measurements are invaluable if one wants to build a predictive molecular model of a collagen fibril or wants to rationally design collagen-based materials with specific mechanical responses. For the most part, available datasets of this kind come from tissue-level mechanical testing combined with X-ray scattering to follow average fibril-level structural changes during deformation^5–8^. Heterogeneity in the failure characteristics of fibrils limits the use of this approach for understanding structure-function relationships when applied tension exceeds the material’s threshold for damage. As an alternative, a few groups are currently developing nano-scale tensile testing at the single collagen fibril level followed by post-testing structural analysis whenever possible^9,10^. This approach has the advantage of directly addressing the fibril-to-fibril differences that may exist both within and between tissues.

**Figure 1:**
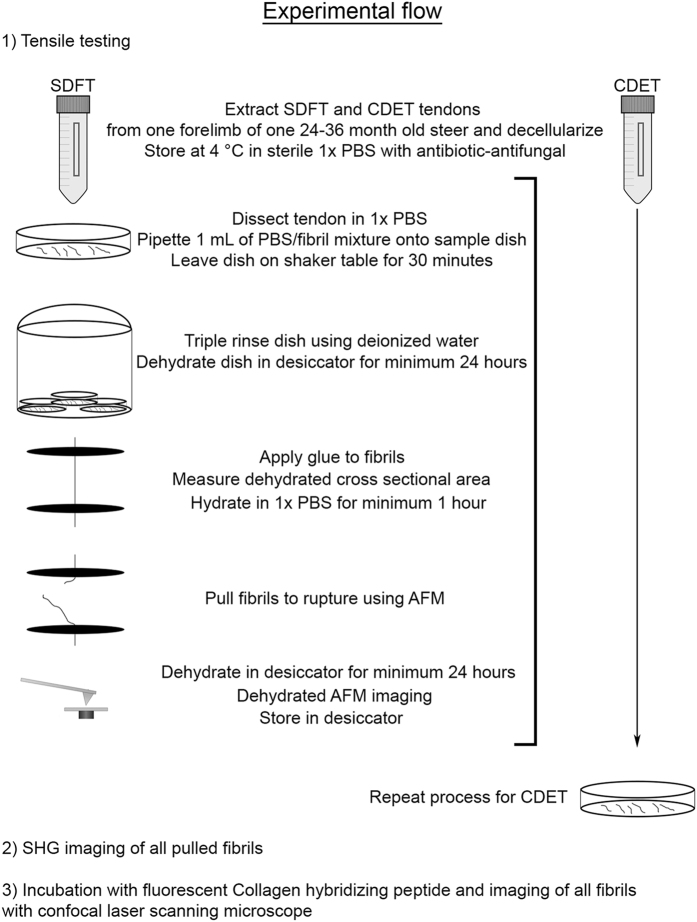
Experimental workflow.

Our approach combines a single fibril tensile testing method with three different imaging techniques, atomic force microscopy (AFM), second harmonic generation microscopy (SHG), and fluorescence microscopy after incubation with a peptide probe that binds specifically to denatured collagen molecules^11^. Each fibril is first stretched to failure then imaged sequentially by AFM, SHG, and fluorescence microscopy (Fig. 1). From these measurements we obtain a stress-strain curve for each fibril, quantify the spatial frequency of plastic damage sites along the fibril using AFM, measure the average SHG intensity and characterize the distribution of the anisotropy parameter along the fibril (a measure of molecular order), and via fluorescence microscopy provide a qualitative yes/no answer to the presence of denatured collagen in the broken fibrils. With this workflow, it is possible to perform fibril-level mechanotyping of collagen-rich tissues as a function of anatomical location, animal source, age, and pathology. The dataset presented here illustrates the utility of this approach: we have been able to demonstrate that tendons exposed to different stress levels *in vivo* are composed of fibrils with distinct structure-function relationships, having both different stress-strain responses and different susceptibilities to structural disruption on overload^11^.

## Methods

### Collagen fibril samples

The collagen fibrils used for this dataset were extracted from superficial digital flexor (SDF) and common digital extensor (CDE) tendons dissected from two forelimbs, each from a different 24–36 month-old steer killed for food at a local abattoir (Oulton’s Farm, Nova Scotia, Canada). Prior to fibril extraction, each tendon went through a decellularization treatment procedure following the method of Ariganello *et al.*^12^. After this step the tendons were kept in sterile phosphate buffer saline (PBS) with 1 % antibiotic/antimycotic solution containing penicillin, streptomycin, and amphotericin B at 4 °C until use. Fibrils were freed from each decellularized tendon into room temperature PBS using a razorblade and metal tweezers. Aliquots of the collagen fibril-containing PBS solution were they transferred to glass dishes on a linear shaker table operating at 1 Hz, where the fibrils were left to adsorb onto the glass substrate for 30 min. Each dish was then rinsed in water, dried under nitrogen, and desiccated for 24 h.

### AFM cantilever calibration

To calibrate the board shaped cantilevers used for tensile testing, we first measured their length (L) and tip height (H, including half of the board thickness) by scanning electron microscopy (Phenom G2 pro, Phenom-World, Netherlands). Then the vertical spring constant K_z_ (N/m) was calibrated by first measuring the vertical deflection sensitivity S_z_ (nm/V) of the cantilever on a Sapphire substrate followed by a fit of the thermal noise spectrum of the cantilever^13^. Finally the lateral spring constant K_L_ (N/m) and the lateral deflection sensitivity S_L_ (nm/V) were estimated using the following formula^14^:

$$K_{L}=\frac{2L^{2}K_{z}}{3(1+\nu )H^{2}}$$

$$S_{L}=\frac{3HS_{z}}{2L}$$

where ν = 0.27, is the Poisson ratio for Silicon <111>^15^.

### AFM imaging

We used a Bioscope Catalyst AFM (Bruker, USA) mounted on an inverted microscope (IX71, Olympus, USA) to perform both AFM imaging and tensile testing. For imaging of the dry fibrils before and after tensile testing we used ScanAsyst fluid + cantilevers (Bruker, USA) with a nominal spring constant of 0.7 N/m, a nominal tip radius of 2 nm and an asymmetric pyramidal shape with an average half-opening angle of 18°. The cantilevers were operated in Peak Force Quantitative Nanomechanical Mapping mode at an oscillation frequency of 1 kHz, a vertical tip velocity of 1.2 mm/s and a force set point of 10 nN. Pixel size was 8 nm for all images; raster frequency was 0.5 Hz before tensile testing and 0.125 Hz after tensile testing.

### Tensile testing

Under an upright optical microscope, dried collagen fibrils with straight segments greater than 100 μm long were selected for tensile testing and two parallel strips of epoxy were laid down perpendicular to the fibril axis using a fine-tipped glass rod controlled via a 3D hydraulic micromanipulator. The glued dishes were desiccated for 24 to 72 h. The dried fibrils selected for tensile testing were first imaged by atomic force microscopy (AFM) to measure their cross-sectional area. For each fibril a short 500 nm segment was imaged, an average profile was calculated and integrated to give the cross-sectional area of the fibril. It is likely that this measurement is a slight overestimate of the true cross-sectional area in the dry state.

The dish was then filled with 3 ml of PBS at room temperature and left to rehydrate for 1h. Tensile testing required stiff cantilevers (TAP525A, Bruker, USA) with calibrated lateral spring constants around 3800 N/m in order to extend the fibril segments to failure through their middle in bowstring geometry. For this purpose we used the Nanoman mode of the AFM to draw a path perpendicular to the fibril axis, the tip was brought in contact with the glass at an applied normal force of 15 μN, integral gain was 1.4 and the proportional gain was 5. These values were selected to obtain reliable pulling without the tip hopping over the fibril. The pulling velocity was 1 μm/s while the lateral force on the AFM probe was recorded at 500 Hz and a video of the test was recorded at 20 fps. Force data from the AFM and deformation data from the video recording were synchronized based on the moment at which fibril rupture occurred. Fibril rupture occurred suddenly, with the fibril being present in the video frame directly before rupture and then absent in the very next frame. The point of rupture was similarly identifiable in the force-time data, with a sudden, precipitous fall in load occurring. Using this point and the frame rate of the video, the force and deformation data were aligned. This raw data was used to assemble a stress-strain curve for each fibril^11^. Four mechanical parameters were measured to describe each stress-strain curve: rupture strain, rupture stress, toughness, and high strain elastic modulus. Rupture strain and rupture stress were measured from the last data point prior to the stress abruptly falling to zero. The data points after rupture were removed from the provided stress-strain curves for clarity. Toughness was the integral of the stress-strain curve, evaluated from 0% strain to the rupture strain value. High strain elastic modulus was calculated as the slope of the stress-strain curve for the last 10% strain preceding rupture.

### Post-rupture imaging

**Figure 2:**
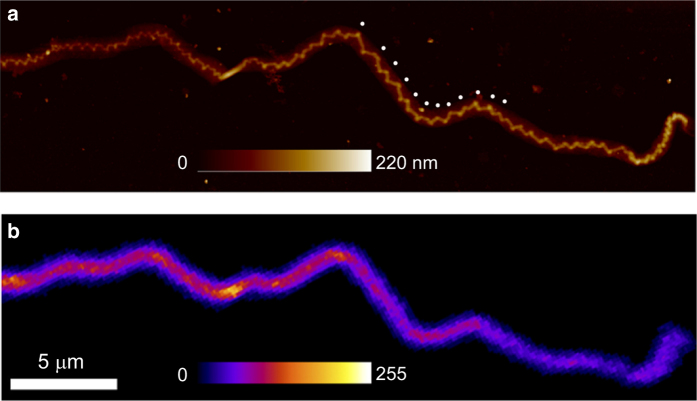
Representative AFM and SHG images of a ruptured CDE fibril. (**a**) AFM image of a dried CDE fibril after rupture. White dots applied by hand mark every second damage site along a portion of the fibril. (**b**) polarization-corrected maximum SHG intensity map of the same fibril.

All ruptured fibrils underwent post-rupture imaging by AFM and then by SHG microscopy. The AFM images were used to measure the average distance between plastic damage sites, which appeared as kinks along each broken fibril (Fig. 2). Every time the fibril centerline showed a sharp bend, this was marked by hand as a kink. The presence and length of any undamaged segments between damaged regions was also registered. For each ruptured fibril a stack of 25 forward scattering SHG intensity images was acquired while the linear polarization of the excitation laser was rotated through [0°–240°] in 10° increments. The maximum forward scattering intensity value at each pixel, selected from amongst the first 18 images in the pol-stack [0°–170°], was used to generate a single polarization-corrected maximum intensity map for each fibril (Fig. 2c)^11^. The field of view of the pol-stacks included both the ruptured and unloaded control portions of each fibril, and an average intensity value was measured for both portions. From each pol-stack we also computed the anisotropy parameter characteristic of the second-order non-linear susceptibility tensor along the fibril.

The SHG response of a collagen fibril, upon excitation by the incident laser beam, is characterized by the second-order non-linear susceptibility tensor (χ^(2)^). Assuming that the fibrils have a cylindrical symmetry (C_6*v*_) and that the Kleinman symmetry is valid in collagen, the non-linear susceptibility of a fibril lying along the x-axis only exhibits two independent tensor components: χ^(2)^_xxx_ and χ^(2)^_xyy_. Therefore, the SHG response of the fibril to the excitation beam, propagating along the z-axis, with a linear polarization within the xy-plane is described by:

$$I_{2\omega }\propto {\left[\rho \cos^{2}(\varphi -\alpha )+\sin^{2}(\varphi -\alpha )\right]}^{2}+\sin^{2}\left(2(\varphi -\alpha )\right)$$

where α and *φ* are the angles of the polarization and the fibril with respect to the y-axis respectively and ρ is the ratio of the two independent tensor components (χ^(2)^_xxx_/χ^(2)^_xyy_). This ratio reflects the anisotropy of the nonlinear response and provides insight into the orientation disorder of the collagen triple helices within the focal volume. To extract the anisotropy parameter from the data, we use the fast-Fourier transform (FFT)-based approach reported by Amat-Roldan *et al.*^16^. To that end, the equation above can be expressed as a sum of Fourier components:

$$I_{2\omega }\propto a_{0}+a_{2}\cos\left(2(\varphi -\alpha )\right)+a_{4}\cos\left(4(\varphi -\alpha )\right)$$

where the three parameters a_0_, a_2_ and a_4_ now contain all the information relative to the tensor element and thus to the anisotropy. As previously demonstrated, the anisotropy parameters can now be determined using:

$$\rho =\sqrt{\frac{a_{4}+a_{2}+a_{0}}{a_{4}-a_{2}+a_{0}}}$$

Glass dishes containing the ruptured fibrils were then treated with a 10 μM solution of fluorescein conjugated collagen hybridizing peptide^17^ (CHP) for 12 h at 4 °C. After incubation, each dish underwent three 5 min rinses with ultrapure water to remove free CHP. Dishes were then dried under argon and subsequently imaged by confocal laser microscopy at an excitation wavelength of 488 nm to reveal the presence of denatured collagen^11^.

## Data records

The combined tensile testing and structural analysis dataset was deposited on FigShare (1). The format, content, and availability of the depositions are described in the following sections.

### Data record 1 – mechanical and post-rupture imaging data

The mechanical quantities obtained from each tensile test are summarized in a worksheet with the physical dimensions of each fibril tested (File 1). The dimensions and the spring constants of the cantilevers used for each tensile test are summarized in a second worksheet (File 2). Each stress-strain curve is also provided (File 3). The results of post-rupture imaging are summarized in a worksheet (File 4), and the raw data for each imaging modality are provided in Files 5 to 7.

**File 1. Tensile data.xls**

COLUMN A – Animal number (1 or 2)

COLUMN B – Tendon type: common digital extensor (e) or superficial digital flexor (f)

COLUMN C – Fibril number

COLUMN D – Length in micrometers of prepared fibril segment before pull-to-rupture

COLUMN E – Dried fibril segment cross-sectional area in micrometers squared

COLUMN F – Cantilever number

COLUMN G – Rupture strain in percent

COLUMN H – Rupture stress in Mega Pascal with respect to the dry cross-sectional area of the fibril

COLUMN I – Toughness in Mega Joules per meter cube obtained by integrating the stress-stain curve (File 3)

COLUMN J – High strain modulus in Mega Pascal obtained by linear least-square fit of the last 10% of the stress-strain curve (File 3)

COLUMN K – Error on the high strain modulus in Mega Pascal obtained by linear least-square fit of the last 10% of the stress-strain curve (File 3)

**File 2. Cantilever spec sheet.xls**

COLUMN A – Cantilever number

COLUMN B – Cantilever manufacturer

COLUMN C – Cantilever model

COLUMN D – Cantilever type

COLUMN E – Cantilever material

COLUMN F – Cantilever length in micrometers

COLUMN G – Tip height in micrometers

COLUMN H – Vertical spring constant in Newton per meter

COLUMN I – Lateral spring constant in Newton per meter

**File 3. Stress strain curves.zip**

Stress-strain curve for each fibril as a two column tabulated text file. The first column is the strain in percent and the second column is the stress in Mega Pascal measured as the tension divided by the dried fibril segment cross-sectional area (File 1, COLUMN E). Each file is named using the first three columns of File 1, for example: animal 1, common digital extensor tendon, fibril 1 is 1e1.

**File 4. Post-rupture imaging data.xls**

COLUMN A – Animal number (1 or 2)

COLUMN B – Tendon type: common digital extensor (e) or superficial digital flexor (f)

COLUMN C – Fibril number

COLUMN D – Rupture location, at the glue or in the middle of the segment

COLUMN E – Average dry fibril height before rupture in nanometers

COLUMN F – Average loss of dry fibril height after rupture in nanometers, standard deviation of the measurement is 2 nm

COLUMN G – Normalized loss of dry fibril height in percent

COLUMN H – Total number of plastic damage sites

COLUMN I – Average spacing of plastic damage sites (kinks) in nanometers, Common Digital Extensor fibrils only

COLUMN J – Ruptured fibril is fluorescent above background level, Yes or No

COLUMN K – Average maximum forward SHG intensity ratio: ruptured segment/unruptured segment

COLUMN L – Peak of the distribution of anisotropy parameter ρ characteristic of the second-order non-linear susceptibility tensor along the ruptured fibril

COLUMN M – Full-width at half-maximum of the distribution of anisotropy parameter ρ characteristic of the second-order non-linear susceptibility tensor along the ruptured fibril

COLUMN N – Peak of the distribution of anisotropy parameter ρ characteristic of the second-order non-linear susceptibility tensor along the unruptured fibril reference

COLUMN O – Full-width at half-maximum of the distribution of anisotropy parameter ρ characteristic of the second-order non-linear susceptibility tensor along the unruptured fibril reference

**File 5. AFM images.zip**

Representative raw AFM images of the dried ruptured fibrils in their original Bruker nanoscope file format. The images were acquired using the Peak Force Quantitative Nanomechanical Mapping mode and can be opened using the freeware Gwyddion (www.gwyddion.net). The first channel is the height, the second channel is the peak force error, the third channel is the DMT modulus, the fourth channel is the logarithm of the DMT modulus, the fifth channel is the adhesion, the sixth channel is the deformation and the seventh channel is the dissipation (http://www.nanophys.kth.se/nanophys/facilities/nfl/afm/icon/bruker-help/Content/PeakForceQNM/Operation/Channels.htm). Only channels 1 and 2 are useful, as the probes used for these measurements were not calibrated to extract quantitative mechanical data. Each file is named using the first three columns of File 1, for example: animal 1, common digital extensor tendon, fibril 1 is 1e1.

**File 6. SHG pol-stacks.zip**

Raw SHG forward intensity pol-stacks containing 25 grey scales images in TIFF format. Each file is named using the first three columns of File 1, for example: animal 1, common digital extensor tendon, fibril 1 is 1e1. The pol-stack of the corresponding unruptured fibril segment used as a reference is indicated with the small letter c at the end of the file name. The field of view is 100 by 100 micrometers for each image and the pixel size is 200 nm.

**File 7. Fluorescence images.zip**

Raw fluorescence images in .lsm format (Carl Zeiss) containing two channels: brighfield and fluorescence. Images can be opened using ImageJ (https://imagej.nih.gov/ij/). Each file is named using the first three columns of File 1, for example: animal 1, common digital extensor tendon, fibril 1 is 1e1. The first file for each fibril is an overview including the unruptured fibril reference for comparison purposes; the other files are region of interests indicated with capital letters, A, B, C as appropriate. The overview images were acquired at a laser intensity of 12 mW except for fibrils 2f1 to 2f11 where the laser intensity was 6 mW. Similarly the regions of interest images were acquired at a laser intensity of 16.5 mW except for fibrils 2f1 to 2f11 where the laser intensity was 7.2 mW. All the images provided are raw and were not treated in any way.

## Technical validation

**Figure 3:**
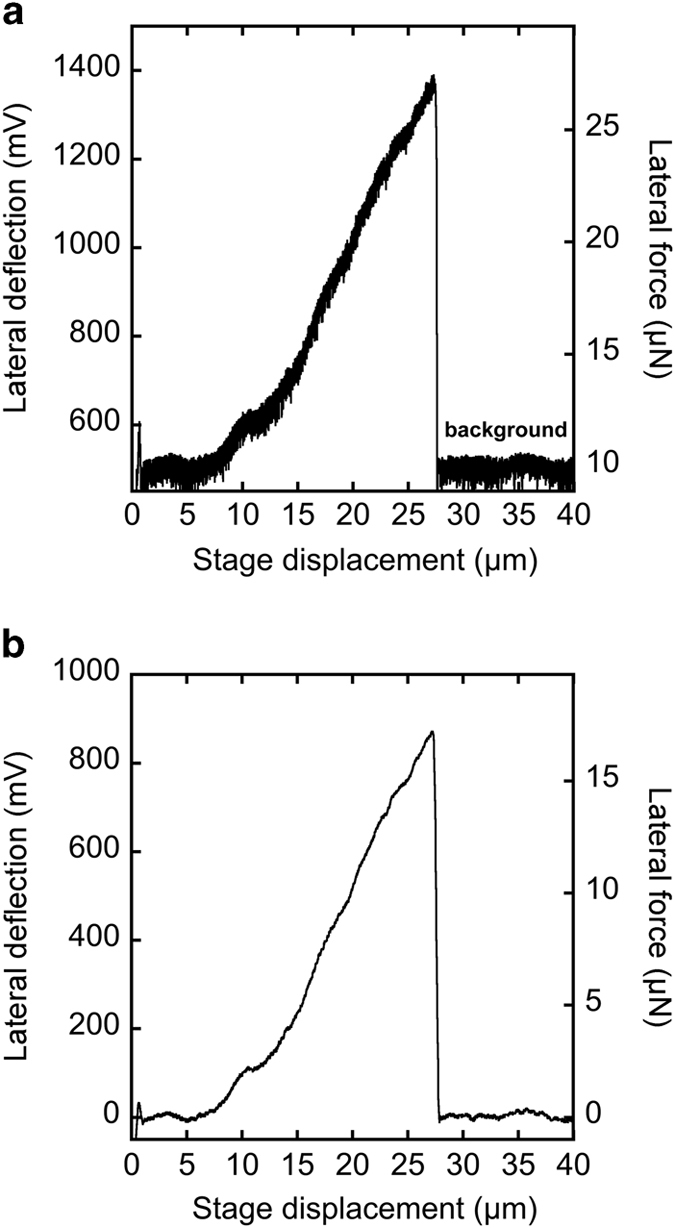
Representative CDE fibril force displacement curve. (**a**) Force displacement curve collected at 500 Hz. In the regime following fibril rupture, the average force value (labeled “background”) was subtracted, removing the force background. (**b**) Force displacement curve after adjacent averaging over 5 points to smooth the curve, and background subtraction.

All the tensile testing modalities used on single collagen fibrils so far suffer from stress localization at points of load application, which means that the rupture strain is underestimated. For this data set we used the bowstring geometry where stress localization occurs both at the glue strips and in the vicinity of the AFM probe. In addition the force applied to the fibril is measured with respect to the background frictional force between the AFM tip and the glass substrate. That frictional force appears as a constant noisy level in the raw data that can be easily filtered and subtracted (Fig. 3). Still because the glass surface is not perfectly clean, the tip may pick-up or may squish particles or macromolecules that were extracted from the tendon at the same time as the fibrils. These events are, we think, responsible for the stress-strain curves not being smooth.

Another specificity of the bowstring geometry is that the fibril is first loaded in bending before reaching full tension. According to Yang *et al.* who performed three point bending experiments on fibrils suspended across a channel, the bending modulus of a single collagen fibril measured in buffer is between 0.07 and 0.17 GPa^18^. Using the upper bound for the bending modulus, a typical fibril radius of 100 nm, a typical prepared fibril segment length of 50 μm and a typical bending deflection of 5 μm before the fibril reaches full tension, we can estimate an upper bound for the bending force in the order of 0.1 nN^18^, several orders of magnitude smaller than the tensile forces we measure which are in the order of 1–10 μN (Fig. 3). In order to present our stress-strain curves in a way that is directly comparable to previously published data, we use the video recording of each test to identify when the fibril is in full tension and ignore force data measured prior to that point as it is a mix of bending and stretching forces. This is why the stress-strain curves we provide (File 3) have a gap between 0% and typically 5% strain. As such these curves should not be used to extract a linear modulus over this strain range. For estimating fibril toughness, we simply extrapolate the data linearly to 0% strain. Because individual fibrils show little “toe” region^19^, the error introduced by this approximation is negligible. Finally all the stresses are measured with respect to the cross-section of the dried fibril before tensile testing. Studies have shown that fibril radius increases by a factor of approximately 2 from the dry state when immersed in buffer^20,21^, so a factor of ~4 can be used to estimate the mechanical properties of fibrils based on hydrated cross-sectional area using our stress data. At this point there is no consensus estimate for the poisson ratio of a fibril immersed in liquid with recent reports of apparent poisson ratio above 1^22,23^, meaning that true stresses can not be estimated easily.

## Usage notes

This stress-strain curve dataset is directly comparable with recently published tensile testing results on single collagen fibrils extracted from different tissues and exposed to different chemical treatments^24,25^. We hope this submission will encourage other research groups to share their datasets as such a collection of stress-strain curves would serve as a unique benchmark for the development of new nano- or micro-fibers for tissue engineering applications. In addition the structural dataset will be useful to researchers studying mechanical damage in collagen-rich tissue.

## Additional information

**How to cite this article:** Quigley, A.S. *et al.* Combining tensile testing and structural analysis at the single collagen fibril level. *Sci. Data*. 5:180229 doi: 10.1038/sdata.2018.229 (2018).

**Publisher’s note:** Springer Nature remains neutral with regard to jurisdictional claims in published maps and institutional affiliations.

## Supplementary Material
